# Navigating safety in practice: a qualitative study of how health care personnel organize medication management in Norwegian home care services

**DOI:** 10.1186/s12913-026-14761-2

**Published:** 2026-05-20

**Authors:** Thomas Asmelash Habtu, Aud Uhlen Obstfelder, Janne Myhre, Nina Beate Andfossen

**Affiliations:** 1 Department of Health Sciences in Gjøvik, Faculty of Medicine and Health Sciences, Norwegian University of Science and Technology (NTNU), East NTNU, Center for Care Research, Teknologivegen 22, Gjøvik, 2815 Norway; 2 Department of Health and Nursing Science, Faculty of Social and Health Science, Inland Norway University of Applied Sciences, INN University, Elverum Norway, Norway; 3 Norwegian Centre for Violence and Traumatic Stress Studies (NKVTS), Oslo, Norway

**Keywords:** Ethnography, Healthcare, Home care services, Medication management, Norway, Patient safety, Professionals, Regulations and guidelines, Qualitative research

## Abstract

**Background:**

Medication management for older adults receiving municipal home care services involves daily coordination between home care staff, general practitioners, pharmacies, and hospitals across frequent care transitions and regulatory requirements. Although regulations, guidelines, and safety approaches are intended to support safe medication practices, healthcare professionals (HCPs) in home care work under conditions marked by time pressure, limited resources, and frequently changing medication information. However, there is limited knowledge about how HCPs interpret and apply these regulatory frameworks in everyday home care practice. This study aims to explore how HCPs organize medication management to ensure patient safety in Norwegian home care services.

**Methods:**

A focused ethnographic design was employed in three purposively sampled Norwegian municipalities. Data were generated through 218 h of participant observation, informal interviews, photographs, and document collection involving 25 participants from various professional backgrounds. The data were analyzed via an iterative ethnographic method inspired by Roper and Shapira.

**Results:**

The findings revealed an overarching cultural orientation, “the here and now,” along with two interconnected pattern categories: “performing resilience: making the day run” and “enacting system integration: chasing the right information.” Medication management was organized through continual coordination, task prioritization, and repeated efforts to obtain and verify medication information. Although HCPs adhered to regulations and guidelines, written procedures were inconsistently referenced in daily work. Instead, medication safety relies on locally organized practices, shared understandings, and adaptation to immediate demands.

**Conclusion:**

Medication safety in municipal home care is maintained through a continuous, interpretive process shaped by the practical realities of daily work. Regulations and guidelines function alongside locally organized coordination and information work rather than as linear instructions. Understanding medication management as it unfolds in the here and now provides insight into the gap between work-as-imagined and work-as-done. This study contributes to the analytical concept of “here and now” to explain how medication safety is enacted through real-time coordination and information work in municipal home care. These findings indicate that guidelines and digital systems require improvement to better support real-time coordination and information work, and safety interventions also need to address local sensemaking and cross-organizational communication.

**Supplementary Information:**

The online version contains supplementary material available at 10.1186/s12913-026-14761-2.

## Background

The use of prescription medications among older adults is increasing worldwide [1, 2]. Medication is a common and vital means of managing both acute and chronic health conditions and maintaining health [3]. However, medication-related harm continues to be a major patient safety concern [4]. Patient safety refers to the absence of preventable harm or adverse events during patient care by healthcare professionals (HCPs) and within the healthcare system [5]. The World Health Organization’s (WHO) global patient safety initiative “Medication Without Harm,” launched in 2017, aimed to reduce medication-related harm by 50% within five years [4]. Although the 2024 WHO report suggests that awareness has likely increased, the target was not fully achieved, underscoring the persistent need to reduce medication-related harm, particularly among older adults [6].

This challenge is intensified by demographic shifts, including an aging population with multiple chronic conditions and an increasing number of individuals aged 65 and older living at home [7–10]. These trends have heightened the importance of safe medication management in home care settings. Managing medications in the home care environment involves complex interactions among various HCPs, institutions, and digital systems [11]. Although the exact rate of medication-related injuries in municipal health and care services is unclear, research shows that 5 to 10% of all hospital admissions are due to improper medication use [12]. Injuries related to medication can occur at any stage of the management process, especially when responsibility is shared among institutions or during care transitions, where medication information may be missing or incomplete [13]. Studies indicate that fragmented communication, differences in professional skills, a lack of standardized routines, and a patient safety culture characterized by fear of blame further increase risks and hinder learning from errors [14–18]. Additionally, polypharmacy raises the risk of medication discrepancies, errors, and adverse events [19, 20]. Overall, these challenges create a vulnerable environment for patient safety among older adults receiving municipal home care.

In this context, national healthcare systems such as Norway’s home care play a central role in addressing the needs of an aging population, a role reinforced by policy initiatives such as the 2018 reform *“A full life – all your life”* [21]. In 2022, 27.7% of Norwegians aged 80 and older received municipal home care services [22]. Moreover, municipalities face persistent workforce shortages and difficulties recruiting qualified HCPs [23]. Home care services are staffed by professionals with diverse backgrounds, in which nurses typically hold primary responsibility for medication management, whereas other staff, such as authorized health assistants, administer, observe, and document treatment in accordance with national regulations [24].

Healthcare systems often turn to regulations and guidelines as evidence-based tools to improve quality and safety [11, 25–27]. In Norway, national guidelines for medication management specify legal requirements, supported by interpretive guidance from the Norwegian Directorate of Health and local municipal implementation systems [25]. These regulations aim to guide safety practices; however, HCPs in home care report that guidelines may conflict with professional judgment and the need for situational decision-making. They are also resource-intensive, reduce patient interaction, and shape local safety cultures through informal coordination and locally organized practices [18]. Studies have shown that low adherence to safety measures is influenced by factors such as limited resources, contextual constraints, and HCPs’ knowledge and attitudes, raising questions about how regulatory expectations align with everyday medication management work [28–31].

Against this backdrop, patient safety perspectives in general have shifted from focusing on error prevention (Safety I) to understanding how everyday work succeeds under different conditions (Safety II) [32, 33]. While Safety II has become more prominent, especially in specialized care, home care remains a complex, dynamic, and resource-limited environment where guidelines often intersect with institutional demands. Research indicates that HCPs rely not only on formal regulations but also on tacit knowledge and collective sense-making to navigate daily practice [34]. However, there is limited empirical research on how regulations shape professional judgment and behavior in practice, with regulations frequently emphasizing compliance over contextual judgment and informal coordination [35].

Despite extensive regulation, there is a limited understanding of how HCPs navigate medication management through situated judgment while balancing patient safety principles, regulatory requirements, and guidelines in everyday home care practice. By examining how regulations and guidelines are interpreted and implemented in everyday practice, this study provides an empirically grounded account of how medication safety is achieved in municipal home care under varying conditions. Therefore, this study aims to explore how HCPs organize medication management to ensure patient safety in Norwegian home care services, paying particular attention to how everyday practices unfold within existing safety approaches, regulations, and guidelines.

## Methods

This article is part of a broader ethnographic research project on medication management in Norwegian municipal home care services. The overall project is grounded in interpretivist, practice-based epistemology and examines medication management as situated everyday practice across actors, settings, and institutional arrangements. Analytic approaches across the project were selected on the basis of the nature of the data and the specific research question rather than on a commitment to methodological uniformity.

This study employed a focused ethnographic design to examine how HCPs interpret and apply safety approaches, regulations, and guidelines in medication management to ensure patient safety in Norwegian home care services [36]. Focused ethnography was especially suitable because it enabled an in-depth exploration of medication management practices in municipal home care over shorter periods and included close observation of the topic as a cultural practice, emphasizing the meanings, beliefs, and adaptations that influence HCPs’ daily routines [37].

### Research site

Norway has a total of 357 municipalities, each with a different population size. Each municipality is responsible for its primary healthcare services, such as home care. The study was conducted in three purposively sampled, geographically separated municipalities with varying population sizes and organizational contexts. According to Statistics Norway 2026 estimates, Municipality A has about 33,400 residents, while Municipalities B and C have approximately 6,650 and 5,100 residents, respectively [38].

Depending on their geographic area and population size, municipalities may have one or more home care units. In our study, the municipality with the highest population, Municipality A, has four home care units, while the other two municipalities, Municipalities B and C, have one unit each. The municipality with four units is organized based on the services they provide and its focus. For example, one of the units focuses on persons with dementia receiving home care, while the other units focus on areas such as rehabilitation, etc. The other two municipalities each have a single home care unit that organizes services within teams, such as a dementia team, etc. Home care units generally serve as bases at specific locations where HCPs work. These home bases have medication rooms, meeting rooms, offices, and workstations with computers. Research coordinators and municipal representatives supported project planning and facilitated access to six home care units in the three municipalities.

### Participants

We purposively recruited 25 HCPs from all six home care units across the three municipalities: two HCPs from each of the four units in Municipality A (*n* = 8), and eight and nine HCPs from the units in Municipalities B and C, respectively (see Table 1). The participant HCPs in our study included nurses, healthcare assistants, and assistants, ensuring a diverse range of perspectives and experiences. The overall age span of participants was 21–60 years. Municipality-specific spans were 26–50 (A), 23–60 (B), and 23–50 (C), representing observed minimum and maximum ages rather than the categorical age groups shown in Table 1.

In Norway, HCPs (*helsepersonell*) are defined by the Health Personnel Act (Helsepersonellloven) as individuals who provide health care to patients, including home care recipients with a specific authorization, licensing, or specialized training [39]. Nurses are bachelor-level HCPs with a three-year university-level education and a professional nursing license. Healthcare assistants are high school diploma holders with four years of healthcare vocational training: two years of theory and two years of practice. Healthcare assistants are licensed professionals who work primarily in primary care, including in-home care. Assistants are those who do not possess formal healthcare education. Assistants can be university or high school students who work in primary care and have limited training to perform specific tasks. Such training can include medication management courses that cover administering and documenting medications while assisting home care recipients.

The inclusion criterion was at least 6 months of experience in their current role and direct involvement in medication management. Recruitment was coordinated through information meetings in each municipality, followed by the distribution of study flyers. Interested HCPs contacted the research team directly, after which the research team developed observation schedules.

**Table 1:** Characteristics of the participants and observation hours

Municipality	A	B	C
*N* = 25	8	8	9
Age			
21–30 31–40 41–50 51–60	2 1 4 1	1 7	1 3 5
Education			
Healthcare assistant Nurse Assistant	3 5	3 4 1	3 5 1
Gender			
Male Female	2 6	1 7	2 7
Experience /year/			
0–5 6–10 11–15 16–20 20–30	3 2 2 1	4 2 1 1	6 1 0 0 2
Observation hours = 218	75	75	68

### Data collection

The study is part of a larger research project called MedSafe-Old, which includes multiple work packages. This specific work package examines the perspectives of home care units and HCPs, while other work packages focus on the experiences of care recipients. Therefore, care recipients are not involved in this particular study. However, care recipients have consented to the fieldworker observing HCPs during their home visits.

Data were collected across all three municipalities, with comparable numbers of participants and observation hours in each (see Table 1). This design strengthens the analytical comparison across contexts. The municipalities differ in population size, and these contextual differences were reflected in variations in staffing structures, coordination practices, and information flow in medication management.

Participant observation was the primary method, supplemented by informal interviews, photographs, internal supervision documents, medication management guidelines, and incident reports. Using multiple data sources enabled a richer and more comprehensive understanding of medication management practices as they unfolded in everyday home care work. Ethnographic observation was guided by a semi-structured guide developed specifically for this study, which was reviewed by the research team. It was used to maintain focus on medication-related practices (Supplementary File 1). Fieldwork was conducted between March and June 2024 by the first author, a registered nurse with experience in clinical practice and qualitative research. The first author’s professional background facilitated access to the field and supported a nuanced understanding of medication management practices, while iterative team discussions addressed potential bias.

A total of 218 h of fieldwork were conducted, with 10 shifts observed in each municipality across day, evening, and weekend shifts. The fieldworker has accompanied HCPs to various locations, including patients’ homes, during morning, evening, and weekend shifts. The fieldworker was also present to observe HCPs’ general meetings, morning meetings, report hours, HCPs collecting medication from pharmacies, meetings with general practitioners (GPs), and medication room routines.

Ninety-five photographs of storage systems and medication organizations at patients’ homes and medication rooms were taken. Incident reports for 2023 were collected from each municipality, covering unintended medication-related events, their causes, and categories.

The field notes included chronological descriptions, impressions, and reflections, and were transcribed after each shift. Informal interviews during observations were transcribed verbatim. Notes were organized into observational, methodological, and theoretical categories to enhance reflexivity and analytic clarity [40].

### Data analysis

Building on the observational, documentary, and visual material generated during fieldwork, the following data analysis examined how medication management practices were identified, compared, and interpreted across municipal home care settings.

Data analysis followed a primarily inductive ethnographic approach, inspired by Roper and Shapira, emphasizing iterative movement among observation, reflection, and interpretation [36]. Analysis began during fieldwork through the writing of detailed fieldnotes and reflexive memos, followed by descriptive coding of practices, actions, and situations as they emerged from the data. Initial analytic attention was directed toward practice-close descriptions of how healthcare professionals organized medication management work in everyday home care settings. Through iterative comparisons across observations, documents, and settings, descriptive codes were grouped into recurring patterns. In a later analytic stage, these empirically grounded pattern categories were synthesized into an overarching analytical cultural orientation, referred to as *“here and now*,*”* which captures a shared temporal focus shaping everyday medication management work. This abductive refinement involved interpretive abstraction grounded in the empirical material rather than the application of predefined theoretical frameworks.

All fieldnotes, photographs, and documents were imported into NVivo 14, a qualitative data analysis software for coding and retrieval [41]. The first stage involved descriptive coding, in which short labels were assigned to data segments to capture participants’ practices, statements, and routines. These codes reflected an emic perspective by preserving participants’ own terms and ways of describing events.

The second stage involved pattern identification, in which descriptive codes were grouped into categories that highlighted recurring practices and cultural logics. At this stage, the analysis moved toward an etic perspective, drawing connections between individual experiences and broader medication management processes. Outlier instances that did not fit the dominant pattern were also examined closely, providing contrast and sharpening interpretations.

The final stage involved abstraction, in which pattern categories were synthesized into broader cultural constructs that captured the temporal orientation shaping HCPs’ daily work and the conditions that framed medication management. Throughout the process, the research team engaged in peer debriefing by reviewing coding samples and interpretations. Reflexive notes documented how the first author’s insider position facilitated access to emic meanings while requiring careful etic interpretation. Member checking with participants in each municipality confirmed that the findings resonated with their experiences and accurately represented their perspectives [42]. The analytical process followed an iterative approach, outlining the steps from field observations to conceptual insights that connect ethnographic data to descriptive coding, pattern identification, and abstraction (Table 2).

**Table 2:** Analytical process illustrating the iterative movement from descriptive coding to pattern identification and abstraction, resulting in the overarching theme “here and now”

Analytical stage	Purpose	Data material used	Analytic focus	Output
Stage 1: Familiarization and descriptive coding	To document and describe medication management practices as observed and reported by healthcare professionals	Fieldnotes from participant observation, informal interview transcripts, photographs, documents, incident reports	Close reading of data; coding of actions, statements, routines, interactions, and situations using participants’ own terms	Descriptive codes representing everyday medication management practices (e.g., task coordination, interruptions, information follow-up)
Stage 2: Pattern identification	To identify recurring practices, shared ways of organizing medication management across settings	Coded qualitative material across municipalities and data sources	Comparison and grouping of descriptive codes into broader pattern categories; comparison across cases; attention to similarities, variations, and outliers	Two pattern categories: *Making the day run* and *Chasing the right information*
Stage 3: Abstraction and synthesis	To interpret how identified patterns relate to broader organizational and systemic conditions	Pattern categories, analytic memos, team discussions	Iterative interpretation linking patterns to regulations, guidelines, and safety approaches; movement between empirical material and conceptual interpretation	Overarching theme: The *Here and now*
Maintaining analytic rigor procedures	To enhance credibility and trustworthiness throughout the analysis	Reflexive notes, peer debriefing notes, member checking feedback	Reflexive examination of researcher position; review of coding and interpretations within the research team; member checking with participants	Refined and validated interpretations consistent with participants’ experiences

### Ethical considerations

Ethical approval for this study was obtained from the Sikt, the Norwegian Agency for Shared Services in Education and Research (ID: 312215). The project was also submitted to the Regional Committee for Medical Health Research Ethics North (REK Nord) for assessment (ID: 629685). REK Nord determined that the study does not fall within the scope of the Norwegian Health Research Act (§ 2) because it does not aim to generate new knowledge about health and disease. Therefore, under national regulations, ethical approval from a medical research ethics committee was deemed unnecessary. The study was conducted in accordance with the ethical principles of the Norwegian national research ethics committees.

All participants provided written, voluntary, and informed consent and signed a consent form to participate before recruitment. Information leaflets about the research project were distributed to patients before the observation periods. The participating HCPs received verbal consent from patients to be accompanied by the first author during their visits. The participants were informed that they could withdraw from the study at any time without consequences. Confidentiality and anonymity were maintained throughout the research process.

During the fieldwork, photographs of the medication storage systems, medication rooms, and documentation practices were taken. No images included identifiable patient information. Patients and HCPs were informed in advance about the purpose of the photographs, and verbal consent was obtained before each photo was taken. Images were anonymized and handled in accordance with national data protection guidelines. All procedures were carried out in accordance with the Declaration of Helsinki and relevant national guidelines.

## Results

We identified an overarching theme, the “*here and now*,” supported by two pattern categories: “*performing resilience: making the day run*” and “*enacting systemic integration: chasing the right information*.” Together, these findings describe how HCPs organize medication management in everyday practice, drawing on shared understanding and contextual experiences to coordinate work and respond to the recurrent demands of home care.

The overarching theme “here and now” captures a shared cultural orientation among HCPs toward managing immediate demands, time pressure, staffing challenges, and frequent information updates in everyday medication management practice. This theme represents an interpretive and integrative lens that synthesizes two empirically grounded pattern categories reflecting recurring practices across the dataset. The two pattern categories describe regularities in behavior, interactions, and organizational conditions, offering insight into how medication management is enacted in practice. In brief, “Making the day run” involves task distribution and informal support, while “chasing the right information” focuses on securing and reconciling medication data across GPs, pharmacies, hospitals, patients, and relatives. Together, the two pattern categories and the overarching theme offer a coherent interpretation of HCP’s medication management practice, shaped by shared routines, professional judgment, and coordination among multiple actors and systems. Table 3 shows the overarching theme, the two pattern categories, and the corresponding descriptive codes.

**Table 3:** Descriptive codes, pattern categories, and overarching theme

Descriptive codes	Pattern categories	Overarching theme
- Morning coordination and task allocation - Working under interruptions and time pressures - Staffing, competence, and uneven authorization - Learning, reporting, and variation in practice	Performing resilience: “Making the day run”	The “Here and Now”
- Tracking medication information across systems - Managing instability in digital and organizational systems - Reconciling discrepancies and making sense of decisions	Enacting systemic integration: “Chasing the right information”	

### Performing resilience: “Making the day run”

#### Morning coordination and task allocation

Across the observed home care units, patients are categorized by their service, diagnosis, or geographic area. Home care units organize their daily work using formal digital task lists, with approximately 20 recipients per list for each shift (morning, evening, and weekend). These lists were accessible to each HCP via their work phone and are integrated with the electronic journal system. Each home care unit operated with 10 to 15 lists, and HCPs were usually assigned to the same list across shifts to ensure continuity of care and familiarity with patients.

In addition to digital systems, HCPs relied on physical report books to communicate information across shifts, including the collection of medication from the pharmacy and its delivery to patients. The electronic journal system was used to document patients’ conditions and follow-ups for certain activities, such as injections. The “remember list” (a direct translation of the local term) is equivalent to a to-do list within the electronic journal system.

The observations revealed that home care HCPs dedicated significant effort to safely and efficiently managing medications, both individually and as a team. This was achieved through open communication, coordination, and collaboration, often under conditions of limited resources and unexpected challenges. They managed task assignments, staffing issues, interruptions, reporting procedures, and medication management across different shifts and patients’ homes.

In their daily workflow, HCPs in municipal home care started coordinating their medication management during morning report meetings. Morning meetings in municipal home care are held in person and are central to organizing daily work. Unlike shift handovers, these meetings combined review and coordination activities. At the start of each shift, HCPs logged in to their work phones, accessed their assigned task lists, and simultaneously received verbal reports and additional tasks from unit leaders. Reports were typically read aloud from printed documents, a message book, or directly from digital systems, and included patients’ conditions and follow-up needs. Additional tasks, such as collecting medication from the pharmacy and delivering it to patients, are assigned to HCPs present on the shift through discussion, taking into account their capacity and professional roles.

After the report, a nurse asked the HCPs if they were familiar with the worklist she had received for the day. She emphasized that she did not know how to prioritize the patients since she was not well acquainted with them. One of the HCPs offered a suggestion, but the nurse did not seem satisfied, saying, “I don’t think I will manage this way.” Another health assistant mentioned that she knew the worklist better and offered to help her sort it accordingly, and the nurse accepted. (Observation 2).

Despite the formal structure of task allocation, the organization of work was highly dynamic. Unexpected events such as one or more HCPs’ absences, hospital admissions, or increased care needs frequently required reorganization of task lists. Such reorganization was commonly observed across units. While unit leaders were formally responsible for adjusting task lists, HCPs also played an active and important role in redistributing tasks in response to changing conditions. For example, most complex medication tasks, like injections or home dialysis, are the nurses’ responsibility. When nurses were unavailable, these responsibilities were redistributed, often concentrating complex tasks among fewer nurses and shifting other responsibilities to healthcare assistants and medication-eligible assistants.

A nurse from the day shift came to the report room and informed one of the HCPs about two tasks scheduled for the evening shift: a medication task and a wound care task. The nurse offered some advice on how to perform the procedures and then left the room. Afterward, they finished reading the day shift’s report, and the unit leader began reading from a message book. While receiving the messages, they also discussed who would take responsibility for the additional tasks. Among the HCPs present, there was an assistant without authorization to administer medication, who had a list of patients needing medication assistance. The assistant informed the group, and they started exchanging and assigning patients among themselves. (Observation 12).

Such changing conditions often required HCPs to attend to patients they had not previously cared for or to assume responsibility for an unfamiliar task list. In these situations, HCPs relied on colleagues with prior knowledge and experience in patient-specific tasks, route planning, and patient prioritization on a list. This interdependence was a key feature of HCPs medication management daily work, and central to maintaining continuity and coordination. Across observations, organizing and reorganizing work often leads HCPs to focus on keeping the day running smoothly.

#### Working under interruptions and time pressures

In addition to task coordination, interruptions during medication work are frequently observed. During a single shift, HCPs engaged in multiple tasks that required adherence to procedures. For example, preparing and controlling pillboxes and pill packs in medication rooms were described by HCPs as crucial procedures to ensure accurate and timely medication administration. Frequent interruptions during HCPs’ performance in these tasks were observed. Institutional guidelines recommend labeling the medication room “Do not disturb”; however, this recommendation was inconsistently implemented, and interruptions continued to occur. Nurses reported that they are often interrupted during medication preparations.

The nurse was interrupted several times while I was inside the medication room. She said, “This should not happen, but I was afraid not to respond. Not responding might be taken personally, and even the leadership does not see that whatever the nurses do in the medication room is more important than other tasks” (Observation 10).

These interruptions arose from unexpected patient needs, inquiries from team members, or urgent administrative tasks. Institutional guidelines indicate that HCPs are responsible for, and should be oriented to, medication management guidelines within their scope of practice. The guidelines also reference healthcare personnel law, indicating that HCPs should assess their own abilities and experience and seek assistance from qualified personnel when needed. However, frequent disruptions, impairing consistency, are often associated with discrepancies, confusion, and errors.

A health assistant found a pill pack that started on Thursday instead of Monday and asked the nurses in the medication room why it was arranged that way, but none of them could explain it. One nurse asked if they had placed a filling order for the missing day, and the health assistant replied that they had not. One of the nurses left her procedure to investigate the issue. (Observation 24).

#### Staffing, competence, and uneven authorization

These coordination challenges were further intensified by persistent staff shortages. HCPs and leaders have noted that the shortage of authorized personnel to manage medications has created various challenges. They highlighted increased workloads, task redistribution, and dependence on less experienced staff. HCPs often expressed frustration about their inability to sustain meaningful patient interactions, particularly when time pressures required them to prioritize completing medication tasks. Under these conditions, HCPs face difficulties in maintaining strict routines, including standardized procedures for preparing and administering medications, managing and verifying orders, and documenting observations. They described how they need to work through routines under time constraints to meet daily needs.

During a conversation with the unit leader and other HCPs around a table, they report having difficulty recruiting HCPs with the necessary authorization to administer medication. Additionally, they mentioned that they have open positions requiring authorized HCPs, but they remain unfilled due to a lack of applicants. They emphasized that they are struggling to bring HCPs into their unit. Another HCP, sitting across the table, added that they have relatively good days when they have enough HCPs to administer medication; however, when there are too many assistants without authorization, they become overwhelmed, covering activities that the assistants are not permitted or able to perform independently. (Observation 12).

Differences in experience and authorization further shaped how medication work was distributed and supported among HCPs. Experienced staff often serve as informal mentors and troubleshooters for less experienced colleagues. For example, new or less trained HCPs struggled with managing polymedicated elderly patients and interpreting complex medication schedules. The opportunities for structured professional discussions and on-the-job training varied across the 3 municipalities.

A nurse said that someone at her unit suggested nurses’ meetings, but she has never attended one, and it has never even happened. She mentioned that it would be nice to have regular professional discussions from time to time. She noted the importance of discussing difficult cases where people do things differently, and it would be helpful to share ideas and expertise. She added that they hold a unit meeting where everyone participates, and the agenda can cover a wide range of topics. (Observation 8).

#### Learning, reporting, and variation in practice

In addition to task execution, the HCPs described challenges related to reporting and learning from medication incidents. Institutional guidelines highlight the importance of reporting errors in medication management. HCPs across the three municipalities mentioned using both formal and informal methods to report medication errors and near misses, with varying levels of management support. Incident reports show that a home care unit saw an increase in unintended incident reports after a new leadership initiative encouraged reporting. However, the HCPs expressed hesitancy in reporting incidents due to fear of judgment, punitive actions, or a lack of follow-up and feedback on their reports.

I asked a follow-up question about whether any medication management incidents had occurred in the last few weeks, and the nurse noted that medications can sometimes be forgotten or misplaced. However, the nurse stated that she does not wish to file an incident report against her colleagues, preferring to inform the person directly involved. She also expressed that some colleagues might take it personally if she submits an incident report. (Observation 1).

The incident reports from the three municipalities revealed that *“medication is not* *taken*” was the most common unintended incident reported. HCPs often reflect on incidents in which medications left for patients are found to have gone untaken. For example, a 2023 unintended incident report for medication management in one municipality’s home care unit indicates that most incidents are due to patients not taking the medication. While institutional guidelines state that medication should be administered at the right time, HCPs report that they do not have enough time to wait for some patients to be ready to take their medication. Instead, they instruct patients and proceed to the next patient, assuming that patients will take their medications.

Observations also show that medication management practices, particularly medication preparation, differ across municipalities. For example, in one municipality, HCPs described their practice as aligned with standards that employ a double-control system, in which the procedure outlined in institutional guidelines requires one nurse to independently review what another nurse has prepared when preparing and controlling pillboxes and pill packs. In contrast, their practices vary across the three municipalities.

Two nurses simultaneously prepare and control pillboxes. The process involves including…both nurses (A & B) take tablets from blister packs, bottles, and vials, placing amounts on the table simultaneously, then Nurse B fills the pillbox compartments accordingly. Nurse A watches as Nurse B puts medications into the pillbox compartments and continues selecting tablets. After finishing the weekly pillboxes, one nurse (sometimes A, sometimes B) stacks them together and stores them in a large container. Nurse A then documents the process in the system, and the second nurse notes in the mobile system and completes the task. They say that they were applying double-controlling mechanisms to ensure that pillboxes were prepared correctly. (Observation 2).

Despite being aware of regulatory expectations, HCPs reported limited access to written medication management guidelines and relied instead on their experience and communication with colleagues to manage daily medication tasks. They use available resources to meet daily goals, complete patient lists, and strive to avoid delays that could affect patients’ medication schedules. While daily task coordination was essential for maintaining workflow, much of this effort depended on access to accurate, timely medication information.

### Enacting systemic integration: “Chasing the right information”

#### Tracking medication information across systems

Across the field sites, observations indicate that medication management in municipal home care involves HCPs’ continuous efforts to obtain, update, and coordinate medication information among multiple actors across different systems. Regulations and institutional guidelines address specific settings and tasks. However, HCPs coordinate the flow of updated medication information between actors and institutions.

HCPs in home care allocate a substantial portion of their time to identifying accurate, up-to-date medication information to ensure timely medication management. Sometimes, they must interfere with other actors’ systems to ensure that the medication process runs smoothly, drawing on their experience.

On the way to the next patient on the list, the nurse called the pharmacy and ordered medications to be picked up before lunch. She placed an order for approximately four patients and told the pharmacy that she would collect it before noon. While she was on the phone, the pharmacist checked the patient’s prescription and told the nurse that the patient did not have a prescription for that specific medication. The nurse replied that if the pharmacist scrolls down a little on the first page of the patient’s prescription, he will find the medication she wants to order. A few seconds later, the pharmacist said he found it and apologized for not seeing it at first. The nurse said she had experienced that before, and everything was alright. The order was placed. (Observation 11).

HCPs reported that they often manage patients on multiple medications who need frequent prescription updates. Medication information is often revised during care transitions or after changes are made by different providers. HCPs said that they regularly need to monitor these changes to keep medication information accurate.

The nurse received an email from a hospital diabetes specialist regarding an increase in the patient’s diabetes medication dosage and forwarded the message to the patient’s GP so that the GP could update the medication list and send it to the pharmacy. I then asked the nurse whether the doctor had already sent the information directly to the GP, and the nurse replied that she believed they had, but it might take some time for the GP to return to them or to send the information to the pharmacy. The nurses mentioned that they preferred to send it to the GPs themselves to expedite the process. She added that she could wait a couple of days to see how it goes, but she chose to send it herself. After that, she wrote a note in the nurse’s book so that others could follow up. (Observation 21).

Moreover, HCPs reported monitoring and coordinating medication information for patients with chronic diseases at home who require frequent care transitions and advanced procedures. They noted that these procedures require experienced nurses and consume multiple hours.

The patient sat on his hospital bed inside a small bedroom filled with medical materials and equipment. The patient informed the nurses that the dialysis machine had stopped working this morning before the antibiotic-induced peritoneal dialysis fluid entered his peritoneum. (Observation 10).

HCPs reported limited opportunities to conduct pretests and prepare for medication reconciliation, as outlined in institutional guidelines. They also mentioned earlier collaboration with nursing students, who aimed to establish a system for working with GPs. However, over time, HCPs said that they could not continue these efforts due to time constraints. Additionally, HCPs noted that patients admitted to hospitals have the chance to review their medications with hospital doctors, as nurses described:

Sometimes I wish that they could be admitted to the hospital so the doctors could review their medication list. I know it is not right to wish like that (Observation 1).

I asked the nurse in the medication room how they handle medication reviews in coordination with the GPs, and the nurse explained that there is very little activity related to patients’ medication reviews. The nurse noted that they needed to take the initiative, and the GP then scheduled a double hour to review patients’ medications. (Observation 19).

#### Managing instability in digital and organizational systems

In searching for the correct medication information, HCPs described navigating fragmented communication channels among municipal home care, hospitals, GPs, and pharmacies, often facing delays and inconsistencies in medication orders and information exchange.

A nurse reported that they had a comorbid patient with very low blood sugar. The nurse said that she had tried to contact the patient’s GP to report the patient’s current condition but had not received a response for more than five days. (Observation 6).

A nurse said that they struggled to receive emails from the hospital about patients’ status and medication changes or that comprehensive discharge reports did not arrive on time after patients’ discharge and home arrival. (Observation 9).

The nurse added that the system is diffuse, making it difficult to know whom to contact, such as a GP or a hospital, regarding a patient and their medications. (Observation 31).

In addition to communication and coordination challenges in obtaining the right medication information, HCPs also noted issues with digital tools, such as technical glitches, unfamiliar interfaces, and steep learning curves.

The nurse mentioned that they are struggling with pharmacy orders since the system transitioned from an analog system, where they had to call the pharmacy to order medications, to a digital system that does not indicate whether a similar order has already been placed. Additionally, they no longer receive a confirmation call or message when they switch to a different pharmacy to prevent duplicate orders. (Observation 31).

#### Reconciling discrepancies and making sense of decisions

**Fig. 1:**
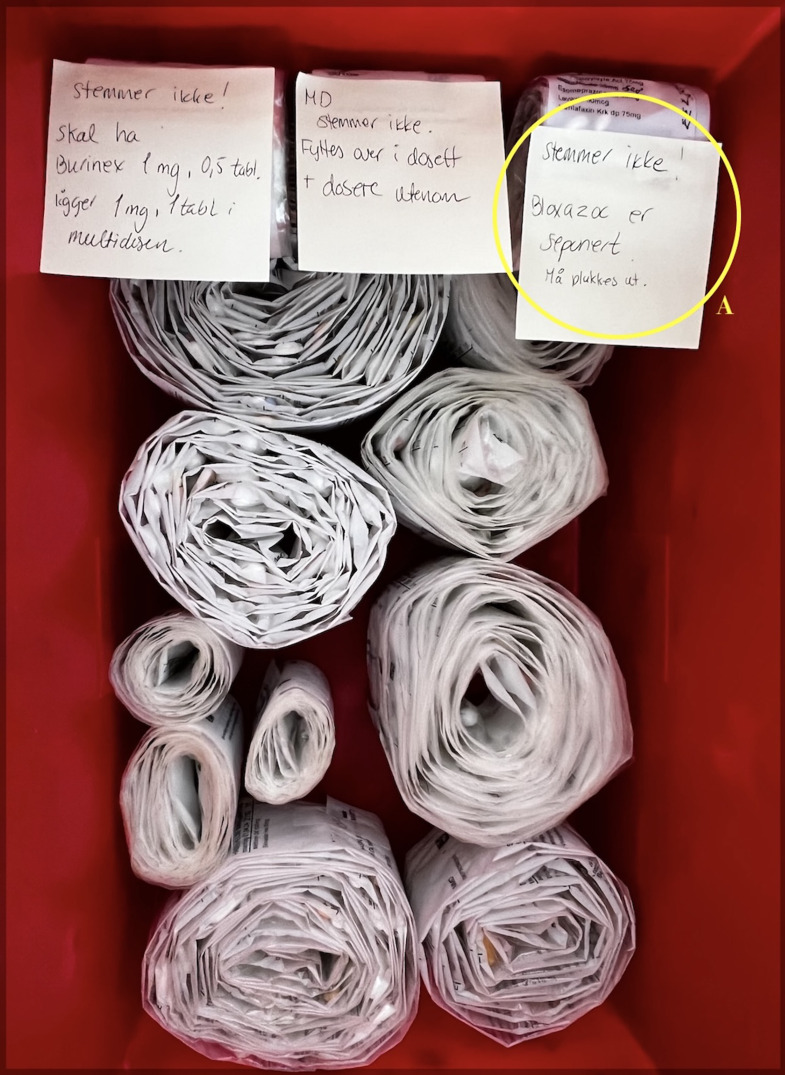
Pill pack rolls were controlled for discrepancies and marked with sticky notes. Photograph by author

HCPs also reported challenges in resolving discrepancies, handling last-minute changes, and ensuring that medications are correctly sorted and labeled before distribution to patients. For example, three of the 13 rolls were found to have discrepancies and required HCP intervention to resolve issues such as (A) “Not correct! Bloxazoc was discontinued. Need to be picked out” (Fig. 1).

The nurse said that the pill pack works perfectly for stable patients; otherwise, it is time-consuming. Pill packs are suitable for patients with stable medical conditions who can take their medication independently. (Observation 9).

The nurse explained that, initially, they do not need to check the pill packs for any change because they believe that the pharmacy has already checked them and that there is no need for a second check. However, the second nurse added that there are always some new changes or prescriptions, so they mostly need to check again. This is mostly because GPs do not submit changes to the pharmacy on time or because they are submitted after the reporting deadline. (Observation 24).

HCPs reported that they often lack explanations for why decisions are made about patients’ medication at different institutions. HCPs said that they are central to the medication management process and are responsible for informing and sharing important information with patients and their next of kin.

After a patient is discharged and arrives home, one of the patient’s medications is discontinued at the hospital, and his wife asks for an explanation. The nurse said that there was very little information about why the hospital changed the patient’s medication. (Observation 8).

In other cases, patients sometimes seek consultations with their GPs without involving HCPs in home care. In such cases, information shared between GPs and patients may not be accessible to HCPs, even though they are responsible for coordinating and managing patients’ medications. HCPs then need to follow up on the updated medication information to coordinate with their unit and the pharmacy.

A nurse reported that some patients contact their GP directly to request changes to their medication. However, the GP did not inform the home care service of these changes or the patient’s updated status. (Observation 9).

Together, these findings describe HCPs’ daily medication management practices, showing how they coordinate and collaborate to complete routine tasks, achieve daily goals, and gather medication information under challenging circumstances. These practices unfold within existing medication management regulations and guidelines in municipal home care.

### The here and now

This section consolidates the two pattern categories, “*making the day run*” and “*chasing the right information*,” by describing how medication management is structured around a shared focus on immediate tasks and priorities in HCPs’ daily work in municipal home care.

The analysis revealed that medication management in municipal home care occurs under daily conditions marked by shifting priorities, immediate demands, and limited predictability. HCPs described focusing on unpredictable tasks and constantly adjusting to meet emergent needs during their shifts. They often respond to new tasks and follow-up activities rather than referring to written guidelines. HCPs reported adapting to their work environment and using available resources to manage daily work and urgent demands. One HCP mentioned that she manages stress quite well, even though the worklist is organized with the same start time for the first four or five patients, as she described:

I don’t see the worklist and stress; I focus instead on the here and now and move on to the next task when I finish this one as soon as possible. (Observation 1).

The observations revealed that HCPs infrequently reference or use institutional medication management guidelines in their daily work. HCPs mentioned that the guidelines are often not accessible or available onsite for consistent use. Instead, HCPs organized their daily work around responding to immediate demands and remained flexible as tasks and responsibilities shifted.

I asked whether they have written routines and procedures for how they practice medication management in the medication room or, in general, in the unit. The HCP I am following pointed her finger toward a shelf above a small work station and said, “That is what we have.” Then, I picked up the file labeled “Medication management procedures,” along with others, and started reviewing it. The other HCP, who was preparing pillboxes at the other station, said, “I’ve never seen this before. I even asked for this document a while ago.” I then asked when the HCP started working at the unit, and the HCP responded, “8 months ago” (Observation 9)*.*

Across the three municipalities, HCPs repeatedly mentioned working in home care settings where priorities and tasks often change. HCPs reported that they need to respond to and adapt to new requirements, such as updated checklists and digital tools, while still managing existing responsibilities that require achieving daily goals.

In a conversation with the HCP on the way to the next patient on the list, the HCP said, “It feels like we need to introduce ourselves to new things all the time. There is always a focus on something new. We have a yearly plan posted in the report room every month; we need to focus on specific areas, such as medication management. Otherwise, we focus and use our energy to go through the day.” The HCP emphasized that many things happen every day. (Observation 1).

Observations and informal interviews with HCPs across the three municipalities revealed recurring challenges in managing daily tasks while responding to unpredictable events and constantly changing medication management information. Daily decision-making, task prioritization, and resource allocation were addressed during ongoing work, with a focus on completing immediate tasks. Overall, immediate demands influenced the daily organization of HCPs’ medication-management work, including how regulations and guidelines were enacted in their everyday practice, as described in the previous sections.

## Discussion

This study aimed to explore how HCPs organize medication management to ensure patient safety in Norwegian home care services. By examining medication management as everyday work, the findings show that patient safety is not secured through formal procedures alone but through continuous coordination, adaptation, and prioritization in practice. HCPs manage medications with a shared focus on immediate tasks and shifting priorities, reflected in an overarching orientation to the “here and now.” This orientation integrates the two pattern categories, “making the day run” and “chasing the right information,” and frames how HCPs attend to, interpret, and apply regulations and guidelines in daily practice.

### Making the day run

Our findings show that “making the day run” depends on HCPs functioning both as autonomous professionals and as a community of practice, where medication management relies on shared understanding and locally established routines. These include informal mentoring, task redistribution, and practical methods for maintaining continuity when staff, time, and information are unevenly distributed. In line with Gabbay and May’s account of how evidence is negotiated and internalized in practice, HCPs use experiential knowledge and shared norms to manage competing demands [34]. Relational coordination perspectives similarly emphasize that shared goals, timely communication, and mutual support sustain performance in resource-scarce settings such as home care [43]. In our study, these forms of coordination were central to “making the day run” and were repeatedly used to sustain medication management work under everyday constraints.

In the context of “making the day run,” the results also show that written medication management guidelines were inconsistently referenced in daily practice and sometimes difficult to find. This does not mean that regulations and guidelines were irrelevant; rather, they served as a formal backdrop that intersected with locally organized practice. The HCPs in our study described selectively applying procedures, reordering tasks, or proceeding without full procedural support when time and staffing constraints made strict adherence difficult. This is consistent with research suggesting that regulatory instruments often emphasize compliance and standardization more than the situated judgment, informal coordination, and tacit knowledge that shape frontline medication management work [35]. They also resonate with findings that home care staff may experience guidelines as a resource-intensive task that sometimes conflicts with patient interaction and clinical priorities [18]. Together, these findings show how daily medication management is sustained through locally organized coordination to keep the work moving under everyday constraints.

### Chasing the right information

Information work has emerged as a central component of maintaining patient safety in medication management. Our findings indicate that safe medication practices depend on an accurate, up-to-date medication list, yet the responsibility for maintaining and reconciling this information is distributed across multiple actors and institutions. The HCPs in our study routinely follow up on delayed updates, reconcile discrepancies, and coordinate changes among GPs, pharmacies, hospitals, patients, and next of kin. Similar experiences have been documented in home care and transitional care research, where fragmented information flows and delayed communication create risks of medication errors and impose additional coordination demands on home care staff [14–16].

Moreover, our findings resonate with descriptions of the “invisible” organizational work that sustains care processes and helps work progress amid complex systems [44]. In-home care, such work was not secondary but essential: without continuous information coordination, medication management routines were disrupted, and staff time was diverted from planned tasks to follow-up and corrections. Multiple studies on medication management have similarly shown that HCPs often compensate for system-level gaps by engaging in additional coordination and verification work to maintain continuity and safety [11, 45]. The pattern of “chasing the right information” shows that patient safety in this context depends on cross-organizational coordination and ongoing maintenance of information infrastructures.

### The here and now

The overarching theme “here and now” emphasizes a shared, practice-based orientation to maintaining medication management through collective coordination under time constraints, staffing limitations, and frequent updates to patients’ medication information.

The two pattern categories “making the day run” and “chasing the right information” illustrate how this overarching “here and now” orientation is enacted in everyday medication management practice. The first captures the organization of daily medication tasks through task distribution, informal support, and negotiated responsibility, whereas the second encompasses the work of securing and reconciling medication information among GPs, pharmacies, hospitals, patients, and next of kin. Taken together, these patterns show that patient safety in home care medication management is achieved through ongoing coordination and information work embedded in everyday routines rather than relying solely on written procedures and guidelines.

The HCPs in our study prioritized immediate demands and shifted priorities by coordinating tasks during ongoing work to ensure safe medication management. Similar patterns of coordination and prioritization have been described in studies of home care and other complex care settings, where safe practices depend on teamwork and informal coordination rather than strict procedural compliance [34, 43, 44]. Therefore, the “here and now” orientation helps explain how HCPs interpret and apply safety approaches, regulations, and guidelines in daily medication management. HCPs’ decisions and priorities are not isolated acts but are shared across team interactions, role shifts, and changes in medication information. This clarifies the practical conditions under which regulations and guidelines are enacted and explains why their application cannot be viewed as a simple translation of written instructions into practice.

In this sense, patient safety culture in home care is not officially mandated; rather, it develops through daily coordination, communication, and shared decision-making in the “here and now.” Moreover, “here and now” illuminates the tension between what regulations and guidelines envision and the reality that medication management is a working process, grounded in ongoing coordination and information work embedded in everyday routines.

### The tension between work-as-imagined and work-as-done

The findings reveal a persistent tension between how medication management is conceptualized through regulations and guidelines and how it actually occurs in daily home care practice. In line with Tresfon et al., *work-as-imagined* refers to the assumptions embedded in regulations and guidelines about how medication management should be carried out, whereas *work-as-done* describes how medication management is accomplished in everyday home care practice under variable and resource-constrained conditions [46].

Moreover, regulations and guidelines often assume a standardized, linear process in which information is readily available, roles are consistent, and tasks can be completed without frequent interruptions [47]. However, our study shows that medication management in home care is influenced by variable staffing, interruptions, cross-sector delays, and frequent changes in medication prescriptions. Under these conditions, HCPs must continually prioritize and coordinate medication tasks throughout ongoing work. The “here and now” orientation reflects this gap as a patterned way of organizing work under constrained and shifting conditions rather than as isolated deviations from procedures.

This helps explain why HCPs sometimes experience guidelines as “cookbook-like” and difficult to follow, akin to simple step-by-step recipes in everyday medication management practice [11, 34]. Framing medication management as a controlled sequence of actions may obscure the coordination and resource effort required to do so effectively in a home care setting. In this way, work-as-imagined may underestimate the extent to which safe medication management relies on ongoing negotiations, informal coordination, and compensatory work to bridge staffing and information flow gaps.

Safety perspectives also highlight this tension. The Safety I approach, shaped by efforts to reduce errors and their causes, has influenced the framing of medication safety as error reduction and barrier strengthening [32, 48]. While these efforts remain vital, our study shows that much home care medication work involves maintaining function amid variability, including managing interruptions, resolving discrepancies, and coordinating information across institutions. Safety II perspectives redirect attention toward how work succeeds under different conditions and how resilience is enacted in everyday practice [33, 46, 49]. At the same time, debates about Safety II highlight limitations in operational guidance and evidence, as well as the risks of oversimplifying complex systems [50]. Our findings indicate that home care medication management requires attention from both perspectives: formal procedures and reporting systems remain essential, whereas daily coordination and information management are key to maintaining safe practices when conditions are unstable.

Tension is also evident in incident reporting. While Aljabari & Kadhim highlight the importance of reporting for safe learning, our study reveals variation in reporting practices and, in some cases, hesitation to report [51]. Reporting systems and cultures can obstruct learning when feedback is unclear, usability is low, or reporting is perceived as personally consequential. These variations in reporting practices may also reflect locally situated patient safety cultures, in which concerns about blame, feedback, and responsibility shape how incidents are handled in everyday work. This aligns with concerns in national oversight regarding the limitations of data extraction from reporting systems [52]. It also highlights a gap between policy goals and the actual capacity of reporting mechanisms to support learning. In home care, where interruptions and information gaps are common, the effectiveness of reporting systems depends not only on formal requirements but also on local conditions that influence whether and how incidents are documented and addressed.

Overall, our study shows that HCPs’ application of regulations and guidelines is shaped by the practical conditions of home care, where safety is sustained through continuous coordination, prioritization, and information work in the “here and now.” This does not negate the value of regulations and guidelines; rather, it clarifies how they are enacted alongside locally organized practices that enable medication management to function under everyday constraints. Moreover, this study advances patient safety research by demonstrating how medication safety in home care is achieved through a shared, practice-based orientation and by introducing “here and now” as an analytic concept to explain how medication safety is enacted through real-time coordination and information work in municipal home care.

### Strengths and limitations

This study possesses several strengths. First, the focused ethnographic design, which combined 218 h of participant observation with informal interviews, photographs, and document analysis across three municipalities, enabled a comprehensive understanding of daily medication management practices in Norwegian municipal home care. The inclusion of HCPs with diverse backgrounds and levels of experience offered a broad range of perspectives on how regulations, guidelines, and safety measures are interpreted and applied in practice. Systematic, iterative analysis inspired by Roper and Shapira, the use of NVivo for data organization, and strategies to enhance rigor, such as reflexive memoing, peer debriefing, and member checking, have strengthened the credibility and trustworthiness of the findings.

This study also has limitations. While the study includes multiple municipalities with differing organizational contexts, the findings are context-specific and should be interpreted within the scope of these settings. The first author’s dual role as a nurse and researcher might have influenced both access and interpretation. Although reflexive practices and team discussions were used to reduce this, some interpretive bias may still exist. Additionally, focusing ethnographically on HCPs’ perspectives means that patients’ and next of kin’s views on medication management and safety were not directly examined. Finally, like all qualitative research, the findings highlight patterns and processes rather than providing quantitative estimates of frequency or effect size; therefore, they should be understood accordingly.

## Conclusion

This study shows that medication safety in municipal home care is sustained through a continuous, interpretive process. HCPs are not passive followers of evidence-based guidelines but active participants in safety efforts, drawing on a mix of formal regulations, experiential knowledge, and locally organized practices. There is a consistent tension between the expectations embedded in regulations, guidelines, and safety approaches (work-as-imagined) and the realities of medication management in daily home care practice (work-as-done).

Within this context, HCPs navigate the “here and now” of medication management by responding to immediate demands, described as “making the day run,” and performing information work, described as “chasing the right information.” These practices illustrate how safety is maintained through continuous coordination and adaptation amid limited resources, shifting priorities, and fragmented information. These adaptations do not signal a lack of professionalism but reflect the real-world conditions of medication management in home care. They also point to the development of a locally situated patient safety culture shaped through everyday coordination and decision-making under variable conditions.

The findings underscore the importance of developing safety approaches, regulations, and support systems that better account for how work is organized in everyday practice. Advancing patient safety in municipal home care may require attention not only to compliance with formal procedures but also to the tacit knowledge, coordination work, and institutional arrangements through which medication management is accomplished in everyday settings. This study positions patient safety as a practice-based accomplishment and patient safety culture as emerging locally from everyday coordination and decision-making in municipal home care medication management.

## Supplementary Information

Below is the link to the electronic supplementary material.

Supplementary Material 1

## Data Availability

The datasets generated and/or analysed during the current study are not publicly available due to the presence of potentially identifying personal information, but are available from the corresponding author on reasonable request.

## Abbreviations

HCP: Healthcare Personnel
GP: General Practitioner
WHO: World Health Organization
UN: United Nations

## References

[1] Charlesworth CJ, Smit E, Lee DSH, Alramadhan F, Odden MC. Polypharmacy Among Adults Aged 65 Years and Older in the United States: 1988–2010. GERONA. 2015;70(8):989–95. 10.1093/gerona/glv013 PMC4573668 25733718

[2] Gao L, Maidment I, Matthews FE, Robinson L, Brayne C, on behalf of the Medical Research Council Cognitive Function and Ageing Study. Medication usage change in older people (65+) in England over 20 years: findings from CFAS I and CFAS II. Age Ageing. 2018;47(2):220–5. 29036509 10.1093/ageing/afx158 PMC6037294

[3] Cadel L, Cimino SR, Von Den Rolf T, James KA, McCarthy L, Guilcher SJ. Medication management frameworks in the context of self-management: a scoping review. PPA. 2021 June;15:1311–29. 10.2147/PPA.S308223 PMC8216068 34163148

[4] Donaldson LJ, Kelly ET, Dhingra-Kumar N, Kieny MP, Sheikh A. Medication Without Harm - Global Patient Safety Challenge on Medication Safety. Geneva: World Health Organaization; 2017. p. 14. Report No.: WHO/HIS/SDS/2017.6.

[5] Jachan DE, Müller-Werdan U, Lahmann NA. Patient safety. Factors for and perceived consequences of nursing errors by nursing staff in home care services. Nurs Open. 2021;8(2):755–65. 33570279 10.1002/nop2.678 PMC7877149

[6] World Health Organization. Medication without harm: policy brief [Internet]. Geneva: World Health Organization. 2024. https://www.who.int/publications/i/item/9789240062764. Accessed 20 December 2025.

[7] Kanasi E, Ayilavarapu S, Jones J. The aging population: demographics and the biology of aging. Periodontol 2000. 2016;72(1):13–8. 27501488 10.1111/prd.12126

[8] Melby L, Obstfelder A, Hellesø R. We Tie Up the Loose Ends: Homecare Nursing in a Changing Health Care Landscape. Global Qualitative Nurs Res. 2018;5:233339361881678. 10.1177/2333393618816780 PMC6295756 30574532

[9] United Nations. World population ageing, 2019 highlights [Internet]. New York: United Nations. 2020. http://creativecommons.org/licenses/by/3.0/igo/. Accessed 25 December 2025.

[10] Schmidt-Mende K, Arvinge C, Cioffi G, Gustafsson LL, Modig K, Meyer AC. Profiling chronic diseases and hospitalizations in older home care recipients: a nationwide cohort study in Sweden. BMC Geriatr. 2024;24(1):312. 38570768 10.1186/s12877-024-04796-7 PMC10993481

[11] Lindblad M, Flink M, Ekstedt M. Safe medication management in specialized home healthcare - an observational study. BMC Health Serv Res. 2017;17(1):598. 28836981 10.1186/s12913-017-2556-x PMC5571490

[12] Nymoen LD, Björk M, Flatebø TE, Nilsen M, Godø A, Øie E, et al. Drug-related emergency department visits: prevalence and risk factors. Intern Emerg Med. 2022;17(5):1453–62. 35129789 10.1007/s11739-022-02935-9 PMC9352618

[13] Olsen RM, Devik SA. Legemidddelbruk og Pasientsikkerhet [Internet]. 2016. (En Oppsummering av Kunnskapp; vol. I). https://omsorgsforskning.brage.unit.no/omsorgsforskning-xmlui/bitstream/handle/11250/2415062/Legemiddelbruk%20og%20pasientsikkerhet.pdf?sequence=1%26isAllowed=y. Accessed 26 December 2025.

[14] Lyngstad M, Melby L, Grimsmo A, Hellesø R. Toward Increased Patient Safety? Electronic Communication of Medication Information Between Nurses in Home Health Care and General Practitioners. Home Health Care Manage Pract. 2013;25(5):203–11.

[15] Huemer J, Eriksen L. Teknologi i Samhandlingsreformen: Utfordringer ved implementering av informasjons- og kommunikasjonsteknologii kommunehelsetjenesten. NSF. 2017;7(1):48–62.

[16] Vellonen M, Härkänen M, Välimäki T. Flow of information contributing to medication incidents in home care—An analysis considering incident reporters’ perspectives. J Clin Nurs. 2024;33(2):664–77. 37803812 10.1111/jocn.16896

[17] Escrivá Gracia J, Brage Serrano R, Fernández Garrido J. Medication errors and drug knowledge gaps among critical-care nurses: a mixed multi-method study. BMC Health Serv Res. 2019;19(1):640. 31492188 10.1186/s12913-019-4481-7 PMC6729050

[18] Idsøe-Jakobsen I, Dombestein H, Wiig S. Exploring homecare leaders’ risk perception and the link to resilience and adaptive capacity: a multiple case study. BMC Health Serv Res. 2024;24(1):340. 38486286 10.1186/s12913-024-10808-4 PMC10941597

[19] Scott IA, Hilmer SN, Reeve E, Potter K, Le Couteur D, Rigby D, et al. Reducing Inappropriate Polypharmacy: The Process of Deprescribing. JAMA Intern Med. 2015;175(5):827. 25798731 10.1001/jamainternmed.2015.0324

[20] Amalberti R, Vincent C, Nicklin W, Braithwaite J. Coping with more people with more illness. Part 1: The nature of the challenge and the implications for safety and quality. Int J Qual Health Care. 2019;31(2):154–8. 30476145 10.1093/intqhc/mzy235

[21] Devik SA, Olsen RM, Fiskvik IL, Halbostad T, Lassen T, Kuzina N, et al. Variations in drug-related problems detected by multidisciplinary teams in Norwegian nursing homes and home nursing care. Scand J Prim Health Care. 2018;36(3):291–9. 30139278 10.1080/02813432.2018.1499581 PMC6381529

[22] Statistik sentralbyrå SSB. 2023 [cited 2023 Aug 20]. Sjukeheimar, heimetenester og andre omsorgstenester. https://www.ssb.no/helse/helsetjenester/statistikk/sjukeheimar-heimetenester-og-andre-omsorgstenester. Accessed 10 August 2025.

[23] Helgheim BI, Sandbaek B. Who Is Doing What in Home. Care Services? IJERPH. 2021;18(19):10504. 34639804 10.3390/ijerph181910504 PMC8508197

[24] Helse- og omsorgsdepartementet. Forskrift om legemiddelhåndtering for virksomheter og helsepersonell som yter helsehjelp - Lovdata. 2008; https://lovdata.no/dokument/SF/forskrift/2008-04-03-320. Accessed 10 August 2025.

[25] Helsedirektoratet. Legemiddelhåndtering: Rundskriv [Internet]., Oslo. Helsedirektoratet; 2025. https://www.helsedirektoratet.no/rundskriv/legemiddelhandtering. Accessed 10 December 2025.

[26] Busse R, Klazinga N, Panteli D, Quentin W, editors. Improving healthcare quality in Europe: characteristics, effectiveness and implementation of different strategies. Copenhagen, Denmark: WHO Regional Office for Europe; 2019. p. 419. (Health policy series). 31721544

[27] Fleck JMC, Pereira RA, Silva AEBDC, Gimenes FRE. Adherence to safety barriers in medication administration: patients’ perception. Rev Latino-Am Enfermagem. 2021;29:e3497. 10.1590/1518-8345.5383.3497 PMC8584808 34755778

[28] Mula C. The examination of nurses’ adherence to the ‘five rights’ of antibiotic administration and factors influencing their practices: a mixed methods case study at a tertiary hospital, Malawi. Mal Med J. 2019 June 27;31(2):126. 10.4314/mmj.v31i2.4 PMC6698626 31452845

[29] Vória JO, Padula BLD, Abreu MNS, Correa ADR, Rocha PK, Manzo BF. Compliance to safety barriers in the medication administration process in pediatrics. Texto contexto - enferm. 2020;29:e20180358.

[30] Vaismoradi M, Behboudi-Gandevani S, Lorenzl S, Weck C, Paal P. Needs Assessment of Safe Medicines Management for Older People With Cognitive Disorders in Home Care: An Integrative Systematic Review. Front Neurol. 2021;12:694572. 34539551 10.3389/fneur.2021.694572 PMC8446192

[31] Solberg H, Devik SA, Bell HT, Olsen RM. The art of making the right exception to the rule: Nurses’ experiences with drug dispensing in nursing homes. Geriatr Nurs. 2022;44:229–36. 35240402 10.1016/j.gerinurse.2022.02.019

[32] Kohn LT, Corrigan JM, Donaldson MS. To Err Is Human: Building a Safer Health System [Internet]. Washington, DC. National Academies Press; 2000 [cited 2025 Nov 20]. http://www.nap.edu/catalog/9728. Accessed 20 November 2025. 25077248

[33] Smith KM, Valenta AL. Safety I to Safety II: A Paradigm Shift or More Work as Imagined? Comment on False Dawns and New Horizons in Patient Safety Research and Practice. Int J Health Policy Manag. 2018;7(7):671–3. 29996589 10.15171/ijhpm.2018.24 PMC6037502

[34] Gabbay J, May AL. Evidence based guidelines or collectively constructed mindlines? Ethnographic study of knowledge management in primary care. BMJ. 2004;329(7473):1013. 15514347 10.1136/bmj.329.7473.1013 PMC524553

[35] Browne J, Bullock A, Poletti C, Cserző D. Recent research into healthcare professions regulation: a rapid evidence assessment. BMC Health Serv Res. 2021;21(1):934. 34493260 10.1186/s12913-021-06946-8 PMC8425088

[36] Roper JM, Shapira J. Ethnography in nursing research. Thousand Oaks. Calif: Sage; 2000. p. 150. (Methods in nursing research).

[37] Trundle C, Phillips T. Defining focused ethnography: Disciplinary boundary-work and the imagined divisions between ‘focused’ and ‘traditional’ ethnography in health research – A critical review. Soc Sci Med. 2023 Sept;332:116108. 10.1016/j.socscimed.2023.116108 37531908

[38] Statistikkbanken – SSB [Internet]. Www.ssb.no. 2026 [cited 2026 Apr 8]. Available from: https://www.ssb.no/en/statbank/table/11342.

[39] Helse- og omsorgsdepartementet. Lov om helsepersonell m.v. (helsepersonelloven) - Lovdata. Helse- og omsorgsdepartementet. 2000;LOV-1999-07-02-64:32.

[40] Fangen K. Deltagende Observasjon. Volume 2. Oslo: Fagbokforlaget; 2010. p. 300.

[41] Dhakal K. NVivo. jmla [Internet]. 2022 Apr 26 [cited 2025 Jan 29];110(2). https://jmla.pitt.edu/ojs/jmla/article/view/1271. Accessed 20 January 2025.

[42] Birt L, Scott S, Cavers D, Campbell C, Walter F. Member Checking: A Tool to Enhance Trustworthiness or Merely a Nod to Validation? Qual Health Res. 2016;26(13):1802–11. 27340178 10.1177/1049732316654870

[43] Spitzer EG, Kaitz J, Fix GM, Harvey KLL, Stadnick NA, Sullivan JL, et al. Developing relational coordination: a qualitative study of outpatient mental health teams. adm policy ment health. 2023 July;50(4):591–602. 10.1007/s10488-023-01261-2 PMC9996570 36892721

[44] Allen D. Re-conceptualising holism in the contemporary nursing mandate: From individual to organisational relationships. Soc Sci Med. 2014;119:131–8. 25181472 10.1016/j.socscimed.2014.08.036

[45] Manskow US, Kristiansen TT. Challenges in obtaining and sharing core patient information in norwegian nursing homes and home care services: a qualitative study of nurses’ and doctors’ experiences. In: Medication Safety in Municipal Health and Care Services [Internet]. Cappelen Damm Akademisk/NOASP; 2022 [cited 2025 Apr 20]. pp. 259–77. https://press.nordicopenaccess.no/index.php/noasp/catalog/book/172#chapters. Accessed 20 April 2025.

[46] Tresfon J, Brunsveld-Reinders AH, Van Valkenburg D, Langeveld K, Hamming J. Aligning work-as-imagined and work-as-done using FRAM on a hospital ward: a roadmap. BMJ Open Qual. 2022;11(4):e001992. 36192037 10.1136/bmjoq-2022-001992 PMC9535208

[47] Sharma A, Gamta V, Luthra G. Ensuring patient safety and trust: the critical importance of regulatory compliance in healthcare. J Pharm Res Int. 2023 July 7;35(18):1–15.

[48] Weigmann K. The ethics of global clinical trials: In developing countries, participation in clinical trials is sometimes the only way to access medical treatment. What should be done to avoid exploitation of disadvantaged populations? EMBO Rep. 2015;16(5):566–70. 25851646 10.15252/embr.201540398 PMC4428044

[49] Birkeli G, Lindahl AK, Hammersbøen ÅM, Deilkås ECT, Ballangrud R. Strategies and tools to learn from work that goes well within healthcare patient safety practices: a mixed methods systematic review. BMC Health Serv Res. 2025;25(1):538. 40229754 10.1186/s12913-025-12680-2 PMC11995654

[50] Verhagen MJ, De Vos MS, Sujan M, Hamming JF. The problem with making Safety-II work in healthcare. BMJ Qual Saf. 2022;31(5):402–8. 35304422 10.1136/bmjqs-2021-014396

[51] Aljabari S, Kadhim Z. Common barriers to reporting medical errors. Hsu SH, editor. Sci World J. 2021 June 10;2021:1–8. 10.1155/2021/6494889 PMC8211515 34220366

[52] Norwegian Board of Health Supervision. Patient harm – continued opportunities for Learning and Improvement | Helsetilsynet [Internet]. Oslo: Norwegian Board of Health Supervision. 2025. (Patient harm). Report No.: 2/2025. https://www.helsetilsynet.no/en/publications/report-of-the-norwegian-board-of-health-supervision/2025/patient-harm--continued-opportunities-for-learning-and-improvement/#. Accessed 10 November 2025.

