# Supplementary material for: Navigating safety in practice: a qualitative study of how health care personnel organize medication management in Norwegian home care services
Source: BMC Health Serv Res. 2026 May 20;26:953. doi: 10.1186/s12913-026-14761-2 (PMC13359423; doi:10.1186/s12913-026-14761-2)
Supplement: Supplementary file 1 — Supplementary Material 1 [file 12913_2026_14761_MOESM1_ESM.pdf]

Participant observation guide:

Participant observation is a qualitative research method where the researcher immerses themselves in the study setting to gather firsthand insights and experiences. In the context of the research question about health professionals' experiences and practices of Medicine Management Assistance for older home care recipients. Important points to consider for conducting participant observation:

1. Role Clarification:
   - Observe how health professionals define and understand their roles in providing medicine management assistance to older adults in home care.
   - Please pay attention to their specific tasks and responsibilities in this role.
2. Interaction with Older Adults:
   - Observe how health professionals communicate and interact with older home care recipients when managing medication.
   - Note the tone of communication, empathy, and practical listening skills employed during these interactions.
3. Handling Medications:
   - Watch closely as health professionals handle and administer medications to older adults.
   - Note their adherence to safety protocols, accuracy in dosage administration, and any specialized techniques used.
4. Challenges Faced:
   - Document any challenges health professionals encounter during the medicine management process, such as dealing with resistant or non-adherent older adults.
   - Observe how they address these challenges and adapt their approaches.
5. Collaboration and Teamwork:
   - Participate in or observe interdisciplinary team meetings involving health professionals, caregivers, and other healthcare providers.
   - Note how health professionals collaborate, share information, and coordinate medication management plans.
6. Communication with Families:
   - Observe interactions between health professionals and the families or caregivers of older home care recipients.
   - Please consider how they provide medication management updates, instructions, and guidance.
7. Documentation and Record-Keeping:
   - Document the methods health professionals use to maintain accurate records of medication schedules, dosages, and any changes.
   - Note whether digital tools or paper-based systems are utilized.
8. Cultural Sensitivity:
   - Observe how health professionals navigate cultural differences and preferences while assisting older adults with medicine management.
   - Note any instances of cultural competence or challenges related to cultural sensitivity.
9. Privacy and Confidentiality:
   - Pay attention to how health professionals ensure the privacy and confidentiality of older adults' medical information during the medicine management process.

10. Training and Education:
    - Observe any ongoing training sessions, workshops, or educational opportunities health professionals engage in to enhance their skills in medicine management assistance.
11. Personal and Professional Growth:
    - Document instances where health professionals reflect on their experiences and make efforts to improve their practice based on lessons learned.
12. Innovative Practices:
    - Look for examples of innovative approaches or practices that health professionals may employ to enhance the medicine management process.

Remember, participant observation involves immersing yourself in the field and taking detailed notes on your comments. Maintaining ethical considerations, building rapport with participants, and remaining open to unforeseen insights that may arise during the observation process is crucial.

Types of data:

1. *Primary observations* include:
    - Date
    - Time of day
    - Location
    - Actors present
    - A sequence of events and any interruptions.
2. *Secondary observations* are any statements by others about what you observed.
3. *Experiential data* relates to your own state of mind, emotions, and reflections.
4. *Circumstantial and background data* about the organization, key roles, etc.
5. *Documents: incident reports, institutional guidelines, etc.*
6. *Photographs*
